# Omega-Conotoxins as Experimental Tools and Therapeutics in Pain Management

**DOI:** 10.3390/md11030680

**Published:** 2013-03-07

**Authors:** Heidi E. Hannon, William D. Atchison

**Affiliations:** Department of Pharmacology & Toxicology, Michigan State University, East Lansing, MI 48824, USA; E-Mail: hannonhe@msu.edu

**Keywords:** neuropathic pain, voltage-gated calcium channel, omega-conotoxin, ziconotide

## Abstract

Neuropathic pain afflicts a large percentage of the global population. This form of chronic, intractable pain arises when the peripheral or central nervous systems are damaged, either directly by lesion or indirectly through disease. The comorbidity of neuropathic pain with other diseases, including diabetes, cancer, and AIDS, contributes to a complex pathogenesis and symptom profile. Because most patients present with neuropathic pain refractory to current first-line therapeutics, pharmaceuticals with greater efficacy in pain management are highly desired. In this review we discuss the growing application of ω-conotoxins, small peptides isolated from *Conus* species, in the management of neuropathic pain. These toxins are synthesized by predatory cone snails as a component of paralytic venoms. The potency and selectivity with which ω-conotoxins inhibit their molecular targets, voltage-gated Ca^2+^ channels, is advantageous in the treatment of neuropathic pain states, in which Ca^2+^ channel activity is characteristically aberrant. Although ω-conotoxins demonstrate analgesic efficacy in animal models of neuropathic pain and in human clinical trials, there remains a critical need to improve the convenience of peptide drug delivery methods, and reduce the number and severity of adverse effects associated with ω-conotoxin-based therapies.

## 1. Introduction

Neuropathic pain is a chronic neurologic condition afflicting more than 15% of the population in the United States. For many patients, this form of pain produces severe distress that can disturb or dominate their daily lives [1,2,3]. Poor management of neuropathic pain can give rise to significant social, psychological, and economic consequences, effectively reducing quality of life [4]. Current strategies in pain management include surgical procedures, psychophysical treatments, and pharmacologic intervention. Of the first-line medications, orally administered non-steroidal anti-inflammatory drugs (NSAIDS) and opioids are most commonly prescribed. In some instances, antidepressants and anticonvulsants are also employed [5,6]. Unfortunately, these drugs are not universally effective in pain-afflicted patients; it is estimated that oral administration of current first-line medications fails to provide adequate and sustained pain relief in up to 30% of patients with neuropathic pain [7]. This may be due, in part, to the fact that efficacy of these medications is often limited by the development of tolerance. Even with adequate pain management, long-term use of these drugs, particularly opioids, is associated with potentially intolerable side effects. Because the molecular targets of first-line therapies serve critical roles in both normal physiological function and pathological pain processing, severe side effects are common [4]. Advanced approaches to pharmacologic intervention and alternative routes of administration are widely sought after both to improve the efficacy of pain management and reduce adverse effects [8]. Additionally, there is a growing movement towards developing therapeutics to treat the underlying cause of neuropathic pain states, as opposed to the existing symptomatic management of pain [9]. Hopefully with a better understanding of the pathogenesis of neuropathic pain, drug targets with greater specificity for the pathologic condition will be identified [10].

## 2. Neuropathic Pain

### 2.1. Epidemiology

Approximately 50 million people in the United States currently suffer from chronic, neuropathic pain [11]. Neuropathic pain is broadly defined as pain arising as a direct consequence of a macro- or microscopically identifiable lesion, or an identifiable disease process affecting the somatosensory system. This class of pain presents with clinical signs of spontaneous pain, parasthesia, and mechanical and thermal hyperalgesia or allodynia [12]. Hyperalgesia is characterized as an increased sensitivity, or lowered threshold, to pain, whereas allodynia is pain arising from normally innocuous, non-painful stimuli. Under normal physiologic conditions, pain serves to warn individuals of dangers in the environment through the sense of touch. However, there is no apparent purpose for the heightened sensitivity to noxious or innocuous stimuli associated with neuropathic pain [13]. Though all known etiologies of neuropathic pain are too numerous to list, the vast majority (approximately 90%) of reported painful neuropathies are classified as diabetic, postherpetic, posttraumatic, or iatrogenic neuralgias [14]. Many of these etiologies may present similar clinical symptoms, yet neuropathic pain states often derive from mechanisms unique to the mediating pathologic condition. Conversely, pain-related symptoms vary widely within the same disease etiology and can change over time. In persistent neuropathic pain states, neurons are damaged both by direct and indirect insult; pathologic changes to uninjured neurons are driven by substances released from adjacent, dying cells. Thus, classification of pain on the basis of localization is precluded by neuroplastic changes that do not respect nerve root or cortical territories [12].

### 2.2. Physiology of Pain Signaling

The neurons carrying signals of noxious stimuli are characterized by their axon fiber diameter and conduction velocity: unmyelinated C-fiber nociceptive neurons are the smallest with a diameter of 0.1–1 μm and slowest with a conduction velocity of 0.5–1.2 m/s, as compared to myelinated Aδ-fiber nociceptive neurons with a diameter and conduction velocity of 1–4 μm and 12–36 m/s, respectively. Consequently, the quality of pain carried by the pain-sensing neurons, or nociceptors, differs; activation of the rapidly-conducting Aδ-fibers leads to localized, pricking “first pain”, whereas C-fiber activation results in burning or dull “second pain” with poor localization [15]. 

**Figure 1:**
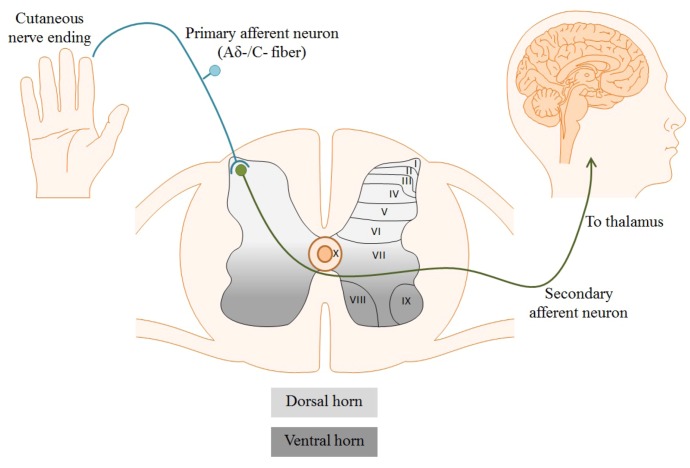
Cutaneous free nerve endings of primary nociceptive afferents detect noxious stimuli in the periphery. Suprathreshold stimuli generate an action potential which propagates towards the nerve terminal in the dorsal horn of the spinal cord. Cell bodies and fibers in the spinal cord are topographically organized in laminae corresponding to function, depicted below as laminae I–X. Aδ-/C-fibers synapse in laminae I and II of the dorsal horn, where primary nociceptors synapse on secondary afferents to transmit pain signals to the brain.

Different classes of nociceptors are activated by distinct forms of noxious stimuli, including mechanical, chemical, or thermal. Regardless of the stimulus, neuronal activation results in its conversion into an electrical signal, referred to as an action potential. Action potentials are generated as a consequence of inherent chemical and electrical driving forces established by ion concentrations in the intracellular and extracellular solutions. Following generation, action potentials propagate along the axons of afferent Aδ- or C-fibers to the nerve terminal in laminae I and II of the dorsal horn of the spinal cord, shown in Figure 1. The laminae are a topographic organization of the cells and fibers within the spinal cord based primarily on sensory modality. Laminae I to IV correspond to exteroceptive sensations; the superficial laminae I and II, specifically, are directly involved in nociception (pain sensation) [15]. Within these superficial layers, nociceptors release pro-nociceptive neurotransmitters, such as glutamate and substance P, to activate postsynaptic dorsal horn neurons. Afferents then follow the spinothalamic tract, sending projections to the dorsal thalamus for processing and perception of painful stimuli [16]. Numerous classes of ion channels and receptors participate in the propagation and processing of pain signals. Among these are voltage-gated Ca^2+^ channels (VGCCs) [17].

## 3. VGCCs and Their Role in Neuropathic Pain

### 3.1. Structure and Function of VGCCs

**Figure 2:**
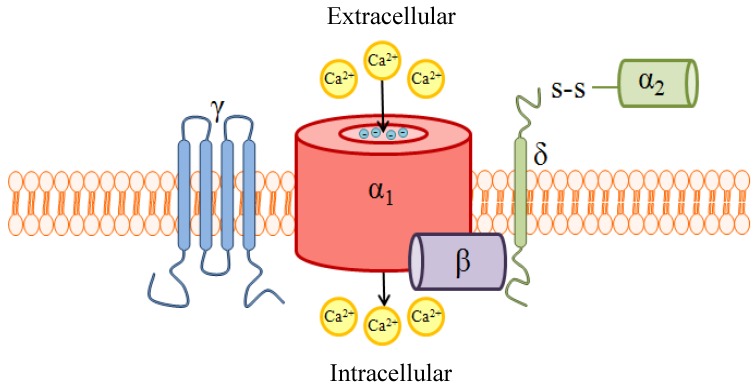
A schematic of high voltage-activated (HVA) voltage-gated Ca^2+^ channels (VGCC) quaternary structure and subunit composition. VGCCs are heteropentameric protein complexes consisting of a pore-forming α_1_ subunit and up to 4 auxiliary subunits. The α_1_ subunit provides the ion permeation pathway, and is thus essential for proper function of the channel. Glutamate residues with the distinct ability to bind Ca^2+^, project into the lumen of the pore and serve as the ion selectivity filter. Additionally, this subunit contains the voltage sensor and drug binding sites. The α_1_ subunit interacts with the cytoplasmic β subunit at a highly-conserved interaction domain; the interaction between these two subunits stabilizes the conformation and promotes trafficking of the α_1_ subunit to the plasma membrane. The β subunit also modulates the activation/inactivation kinetics of the α_1_ subunit. The α_2_δ subunit, comprised of an extracellular α_2_ subunit tethered by the transmembrane δ subunit, enhances expression of α_1_ and also contributes to regulating channel kinetics. The role of γ is not entirely understood, but γ is not required for VGCC formation or function in many tissues. Reproduced with permission from Marrero-Rosado *et al.* [24].

VGCCs, depicted schematically in Figure 2, are heteromeric proteins comprising 5 subunits: the pore-forming α_1_ subunit and the smaller auxiliary β, α_2_, δ, and γ subunits. These channels mediate Ca^2+^ influx into the cell following membrane depolarization, and hence are termed “voltage-gated”. Two distinct classes of VGCCs are generally recognized: the high voltage-activated (HVA) and low voltage-activated (LVA) channels. Each class is characterized by the degree of depolarization required for channel activation, a biophysical property which is largely determined by the α_1_ subunit.

The membrane-spanning α_1_ subunit is arguably the most important subunit of VGCCs, as its expression is required for proper function of the protein. The α_1_ subunit is composed of 4 homologous domains, each with 6 transmembrane segments, which form the pore of the ion channel. Genes encoding ten distinct α_1_ subunits have been identified and are thought to underlie all native Ca^2+^ currents. It is the expression of the α_1_ subunit gene which classifies the VGCC subtype: L-, N-, P/Q-, R-, and T-type (*Ca_v_X.X*). The molecular diversity of the α_1_ subunit, isoform classification, and physiologic distribution of VGCCs is presented in Table 1. In addition to providing the ion permeation pathway, the pore formed by the α_1_ subunit contains the Ca^2+^ selectivity filter and voltage sensor; it is the voltage sensor which confers the voltage-dependence of activation of VGCCs. Binding sites for most pharmacologic antagonists of VGCCs, many of which reduce Ca^2+^ current through direct block of the pore, are also found within the α_1_ subunit. Auxiliary subunits of the VGCC serve to enhance expression, stabilize the conformation and promote membrane trafficking of the α_1_ subunit, and regulate activation/inactivation kinetics of the channel. Because VGCCs are widely expressed throughout the body, particularly in excitable and secretory cells, the function of the channel is largely dictated by its subunit composition [18,19,20]. Diversity of VGCCs can be enhanced further with alternative splicing of mRNA transcripts. Splice variants of VGCCs typically display unique biophysical properties and are often expressed in a tissue-specific manner, suggesting that VGCC expression can be tailored to necessitate distinct physiological functions [21]. Interestingly, perturbations in expression of VGCC splice variants have been linked to pathologic conditions, including neuropathic pain [22,23]. 

Ca^2+^ is a ubiquitous signaling molecule critical to a wide range of physiologic processes in virtually all cell types, including neurons [25]. Because sustained global elevations in intracellular Ca^2+^ (Ca^2+^_i_) can initiate Ca^2+^-dependent cell death, [Ca^2+^]_i_ is tightly regulated by intracellular storage organelles, Ca^2+^-binding proteins, and membrane transporters and ion exchangers. At rest, neuronal Ca^2+^_i_ is maintained at 10–100 nM, but can rapidly rise with neuronal activation and action potential generation [26,27,28]. Ca^2+^ entry through VGCCs following neuronal activation can further modulate membrane excitability and promote action potential propagation along the length of the axon. Within the presynaptic nerve terminal, VGCCs are tightly associated with Ca^2+^-dependent vesicular release proteins. As a result of this coupling, Ca^2+^ influx through synaptic VGCCs can evoke release of neurotransmitter. Additionally, localized elevations in Ca^2+^_i_ can also activate downstream signaling events, such as enzyme activation and gene regulation, through activation of Ca^2+^-dependent effector proteins. Perturbations in Ca^2+^ signaling may therefore result in aberrant electrical activity, neurotransmission and gene transcription [20]. Abnormal Ca^2+^ signaling is implicated as an underlying mechanism of pathogenesis in numerous disease states, including neuropathic pain.

**Table 1:** VGCCs are classified into five isoforms based upon biophysical and pharmacological properties. These characteristics are largely imparted upon the channel by the α_1_ subunit, which is encoded for by discrete *Ca_v_* genes. (* HVA: high voltage-activated; LVA: low voltage-activated).

VGCC Classification	Gene	α_1_ subunit	Voltage activation	Distribution
L-type	*Ca_v_1.1*	α_1S_	HVA *	Skeletal muscle cells
	*Ca_v_1.2*	α_1C_		Neurons, cardiac myocytes, endocrine cells
	*Ca_v_1.3*	α_1D_		Neurons, cardiac myocytes, pancreatic β-cells
	*Ca_v_1.4*	α_1F_		Retinal cells
P/Q-type	*Ca_v_2.1*	α_1A_		Neurons, pancreatic β-cells
N-type	*Ca_v_2.2*	α_1B_		Neurons, pancreatic β-cells
R-type	*Ca_v_2.3*	α_1E_		Neurons, endocrine cells
T-type	*Ca_v_3.1*	α_1G_	LVA	Neurons, cardiac myocytes, smooth muscle cells, endocrine cells
	*Ca_v_3.2*	α_1H_		Neurons, cardiac myocytes, smooth muscle cells, endocrine cells, kidney cells
	*Ca_v_3.3*	α_1I_		Neurons

### 3.2. Ca^2+^_i_ Perturbations in Neuropathic Pain States

The abnormal neuronal activity which underlies symptoms of neuropathic pain, irrespective of disease etiology, includes peripheral and central sensitization, and spontaneous ectopic nociceptor activation [12]. Sensitization is characterized by a reduced threshold for noxious stimuli and increased action potential firing in response to suprathreshold stimuli [29]. Electrophysiologically, these phenomena manifest as a result of alterations in current carried by voltage-gated ion channels, which in turn disrupt neuronal excitability and action potential generation [30,31,32]. Of the VGCCs, evidence indicates strong involvement of the N- and T-type isoforms in nociception under normal physiologic and chronic pain conditions [33,34]. Electrical abnormalities mediated through these VGCCs isoforms present concomitantly with induced pain states in animal models, supporting their critical role in the generation and maintenance of neuropathic pain [29]. Although some studies also suggest roles for P/Q- and R-type VGCCs in the neurotransmission of pathologic pain, these isoforms have not yet been validated as analgesic targets in humans [35,36,37,38,39].

**Figure 3:**
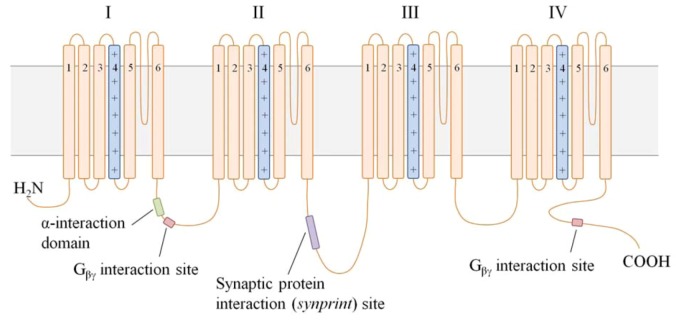
A schematic of the secondary structure of the α_1B_ subunit. VGCC α_1_ subunits consist of 4 domains (I–IV), each with 6 transmembrane segments (S1–S6). The S4 region in each domain (light blue) serves as the voltage sensor, while the membrane-integrated loop between S5 and S6 is believed to form the channel pore. The positively charged glutamate residues necessary for Ca^2+^ selectivity in the pore are depicted (+). The cytoplasmic loop between domains I and II (I–II linker) contains 2 critical interaction sites: (1) the α-interaction domain permits association of the α_1_ and β subunits, and (2) the G_βγ_ interaction site binds G_βγ_ in G protein-mediated channel inhibition. A second G_βγ_ interaction site on the *C*-terminus further modulates channel inhibition. The *synprint* site on the II–III linker tethers the α_1_ subunit to Ca^2+^-dependent vesicular release proteins for rapid release of transmitter following VGCC activation.

All VGCC isoforms are expressed in the dorsal horn of the spinal cord, as demonstrated in immunocytochemical and electrophysiological studies [40,41,42,43,44]. Using subtype-specific antibodies and peptides in immunohistochemical and audioradiographic studies, respectively, expression of N-type channels was shown to be localized to presynaptic nerve terminals of neurons terminating in laminae I and II [45,46]. Voltage-dependence of activation and rate of inactivation of the N-type VGCCs are intermediate, as compared to other VGCC subtypes. In addition to voltage-dependent channel inactivation, Ca^2+^ current through *Ca_v_2.2* VGCCs is uniquely modulated through G protein-mediated events. Because channel modulation via second messengers is not reversed by strong membrane depolarizations, this additional mechanism for limiting *Ca_v_2.2*-mediated Ca^2+^ current is referred to as “voltage-independent inhibition” [47]. Although the exact mechanism is unclear, membrane-delimited G_βγ_ dimers and the cytoplasmic linker region between domains I and II of the α_1_ subunit, shown in Figure 3, are required to reduce Ca^2+^ current [48,49]. The intracellular *N*-terminus of the α_1B_ subunit may provide further determinants for G protein modification of the channel. G_βγ_-bound VGCCs are unable to open, and are only converted into “willing” channels with the dissociation of the G protein. Presynaptic inhibition, via voltage-dependent or -independent mechanisms, limits transmitter release from the nerve terminal and effectively reduces neurotransmission of signals from persistent stimuli [50,51]. Consequently, perception of painful sensations is reduced following presynaptic inhibition of nociceptive neurons in normal pain states [52].

**Figure 4:**
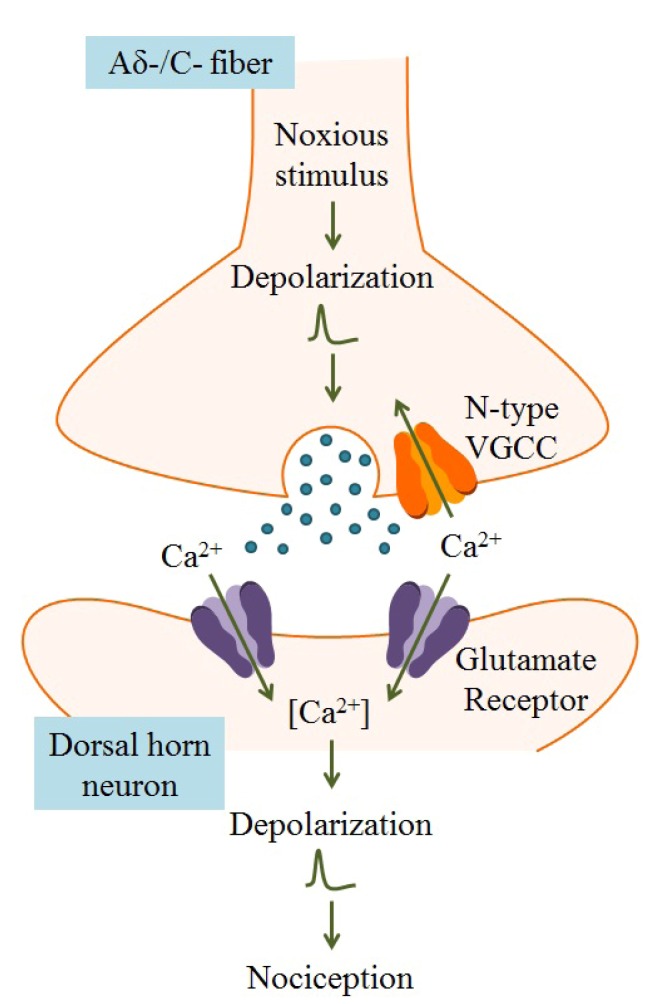
Within the dorsal horn of the spinal cord, arrival of an action potential in the nerve terminal of the Aδ-/C-fiber leads to activation of voltage-gated ion channels, including VGCCs. Because N-type VGCCs are located near neurotransmitter vesicle release sites and are closely associated with Ca^2+^-dependent vesicular release proteins, Ca^2+^ entry through these channels results in release of neurotransmitters, such as glutamate, that activate nociceptive neurons. Postsynaptic ligand-gated ion channels, typically glutamate receptors, are activated by neurotransmitters, leading to membrane depolarization and dendritic action potential propagation. Transmission of action potentials originating from painful stimuli to the brain results in nociception. Adapted from Schmidtko *et al.* [54].

The α_1B_ subunit contains an additional unique interaction site within the cytoplasmic linker between domains II and III. This region, termed the “synaptic protein interaction (*synprint*) site”, permits the association of N-type VGCCs with presynaptic vesicular release proteins. Because neurotransmitter release is a Ca^2+^-dependent process, the tight physical interaction between presynaptic VGCCs and vesicular release machinery is required for optimal neurotransmission [53]. The high density of N-type VGCCs in presynaptic nerve terminals of Aδ-/C-fibers, and association of these channels with intracellular vesicular release proteins imparts a critical role to N-type channels in pain signaling, as illustrated in Figure 4 [42,53]. Furthermore, all *Ca_v_* channels have received attention in neuropathic pain studies for their nearly exclusive expression in the peripheral and central nervous systems. Pharmacologically targeting an ion channel with localized distribution and a role in nociceptor neurotransmission would, in theory, be both an efficacious and safe strategy for better pain management [20].

Indeed, animals lacking *Ca_v_2.2*, the gene encoding the α_1B_ subunit of N-type channels, do not display pain-related behaviors following surgical- or chemical-induced pain states [55,56]. These animal models are standard in pain-related research; neuropathic pain states are often produced in animals by peripheral nerve lesion with spinal nerve ligation or nerve-crush procedures, or by peripheral tissue damage with injection of inflammation-producing chemicals, such as formalin. In these models of pain, animals develop peripheral sensitization which results in neuronal hypersensitivity to mechanical and thermal stimuli [29]. Although surgical- and chemical-induced tactile and thermal hyperalgesia are suppressed in *Ca_v_2.2*^−/^^−^ mice, acute pain states persist [56,57,58]. These *in vivo* studies suggest that N-type VGCCs are more directly involved in chronic nociception, perhaps as a consequence of the channel’s biophysical properties and characteristic slow rate of inactivation [39].

Nerve damage, either by direct lesion, inflammation, or disease conditions, can influence the expression pattern of multiple ion channels, lending a potential explanation to the altered neuronal excitability in neuropathic pain [59]. Expression of N-type channels localized in laminae I and II is enhanced following nerve injury or tissue inflammation in animal studies [44,60]. Consequently, the Ca^2+^ current carried by N-type channels in nociceptors is also increased at the level of the soma [61]. However, because the small size of the nerve terminal precludes measurement of current via conventional electrophysiological methods, it is difficult to determine the functional consequence of increased N-type VGCC expression at the level of the presynaptic nerve terminal. Given the critical role of these channels in vesicular release of transmitter, it is hypothesized that increased expression of N-type VGCCs in neuropathic pain states enhances release of pain-inducing neurotransmitters [4]. This upregulation of N-type channels may also explain the increased potency of isoform-specific VGCC blockers in suppressing tactile and thermal hyperalgesia induced by direct nerve lesion [35].

Interestingly, one splice variant of *Ca_v_2.2* is preferentially expressed in both Aδ- and C-fiber nociceptive neurons, and its level of expression is altered in neuropathic pain states [22,23]. Alternative splicing of exon 37 in *Ca_v_2.2* yields two mRNA splice variants of equal length, namely 37a and 37b. The result of this posttranscriptional modification is an α_1_ subunit that differs in only 14 amino acids at the *C*-terminus [22]. While exon 37b-containing channels are generally more abundantly expressed throughout the nervous system, exon 37a-containing channels are highly enriched in nociceptive neurons [23]. The functional consequence of this splice event is a change in the biophysical properties of the N-type channel and alteration in *Ca_v_2.2* modulation by G proteins; larger whole-cell currents and enhanced voltage-independent inhibition are measurable in nociceptors containing the 37a splice variant [22,62,63,64]. Involvement of exon 37a-containing channels in neurotransmission of pain signals is supported by studies in animal models of neuropathic pain. Interestingly, mRNA transcript levels of 37a-containing channels are reduced by approximately 50% following spinal nerve ligation, while those containing the 37b variant are relatively spared. Although this, in theory, reduces the whole-cell current in nociceptive neurons, it also reduces the extent of voltage-independent inhibition, leaving the activity of N-type VGCCs largely unregulated in pain states, as confirmed in animals lacking exon 37a [23,65]. Unfortunately, it is difficult to develop pharmacologic inhibitors which distinguish between these discrete *Ca_v_2.2* splice variants because the amino acid sequences diverge in a region of the protein which does not bind canonical VGCC antagonists [22,28].

## 4. Targeting VGCCs with Biotoxins in the Management of Pain

### 4.1. The Biodiversity of Conotoxins

Animal venoms have recently received the attention of pharmaceutical development for improved management of many disease states, including neuropathic pain. These poisons have garnered the attention of researchers and medical professionals for their characteristic ability to block critical elements of the nervous or muscular systems, notably ion channels. By harnessing the compounds which potently and specifically block discrete ion channels, there is promise for medicines with greater efficacy and reduced off-target or adverse effects [66]. The ω-conotoxins produced by piscivorous cone snails presently remain amongst the most selective antagonists of N-type VGCCs and, for that reason, are being pursued as drug candidates for the management of neuropathic pain [67,68]. 

*Conus* species are a large genus of gastropodmollusks found with great diversity in the Western Indo-Pacific regions. Although the majority of the 700 species of *Conus* are indigenous to the coral reefs of tropical seas, a subset of species have adapted to more temperate environments. All *Conus* species are believed to be venomous predators, feeding primarily on worms, mollusks and fish. Venoms are synthesized within the tubular ducts of the cone snail and, upon contact with prey, are squeezed through a barbed radula for direct injection into the prey. Particularly within the fish-hunting cone snails, there exists a critical need for venoms to paralyze the prey rapidly. Without rapid and complete paralysis, the violent jerking movements of pierced fish could injure the snail or attract competitive predators [66]. 

Preliminary studies of *Conus* venoms revealed a complex pharmacologic profile suggesting the presence of multiple toxins with discrete actions [69,70]. Indeed, *Conus* venoms comprised a mixture of 100–200 peptides of variable length (9–100 amino acids) which inhibit critical components of the neuromuscular system of the prey. Molecular targets of the conopeptides, or conotoxins, include numerous classes of ion channels, membrane receptors, and transporters [71,72]. Aptly, the toxins derived from *Conus* species are classified by their pharmacologic activity. For example, the α-conotoxins are antagonists of both muscle- and neuronal-type nicotinic acetylcholine receptors, while μ-conotoxins inhibit skeletal muscle voltage-gated Na^+^ channels. Both of these molecular targets significantly contribute to synaptic transmission at the neuromuscular junction. Because α- and μ-conotoxins produce paralysis through block of neuromuscular transmission in all tested vertebrates, the clinical application of these conotoxins is limited. A third class of conotoxins, the ω-conotoxins, produce paralysis in the prey through inhibition of VGCCs in an isoform-dependent manner. Not surprisingly, by blocking VGCCs, the ω-conotoxins inhibit evoked released of neurotransmitters in mammalian *in vitro* systems [23,54,63]. This effect manifests as a reduction in excitatory neurotransmission, as demonstrated in rat hippocampal and spinal cord slices *in vitro* [73,74]. Interestingly, mammals are not susceptible to paralysis following ω-conotoxin poisoning, unlike fish, despite the fact that neurotransmission is effectively blocked [66]. The reason for this is that ω-conotoxin-sensitive ion channels play a minor, if any, role in acetylcholine release at post-developmental mammalian neuromuscular junctions [75].

### 4.2. Use of ω-Conotoxins as Analgesics in Models of Neuropathic Pain

**Figure 5:**
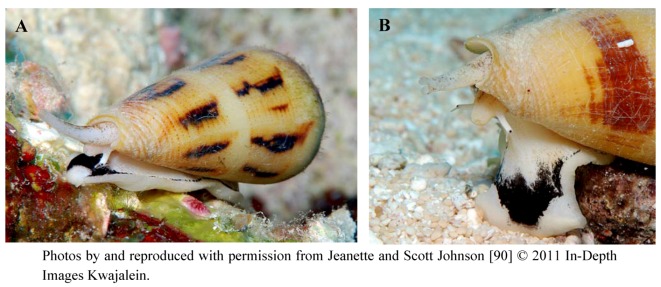
(**A**) Indigenous to regions of the Indian and Pacific Oceans, *Conus magus* synthesizes venom containing a complex mixture of conopeptides to paralyze its prey rapidly. Among this mixture of peptides is ω-conotoxin MVIIA, the conopeptide from which ziconotide is derived. (**B**) Close-up of the barbed radula through which venom is ejected.

Ziconotide, or SNX-111, is the first drug in a class of selective VGCC antagonists derived from *Conus* species [54]. Specifically, ziconotide is a synthetic form of ω-conotoxin MVIIA, a 25 amino acid peptide [76]. The species from which ω-conotoxin MVIIA is isolated, *Conus magus*, is shown in Figure 5. The neuroactivity of ziconotide was confirmed in mammals following intrathecal injection in healthy wildtype mice; animals developed a characteristic persistent tremor within minutes of ziconotide administration. The conotoxin did not elicit any identifiable effect following oral or intravenous administration, likely due to rapid degradation by digestive enzymes and peptidases, respectively, as well as the failure of the peptide to cross the blood-brain barrier [66]. Radioligand binding experiments in rat neocortical neurons and synaptosomes revealed that ziconotide binds rapidly and reversibly to N-type VGCCs with an affinity of 1–18 pM [77,78,79,80]. This binding results in inhibition of N-type channel-mediated Ca^2+^ currents, as demonstrated in multiple types of primary or immortalized mammalian cell cultures that express either native or recombinant channels [81,82,83,84]. The block induced by ziconotide appears to be independent of voltage and the biophysical state of the VGCC (e.g., open, inactivated, closed) [39]. Given the pivotal role of N-type channels in pain signaling, it is not surprising that inhibition of these channels by ziconotide elicits an analgesic effect through the inhibition of transmitter release in nerve lesion and formalin-induced animal models of neuropathic pain [68]. Intrathecal administration of ziconotide produced long-lasting suppression of pain-related behaviors, including tactile and thermal hyperalgesia, in afflicted animals [85,86,87,88,89].

### 4.3. The Clinical Application of Ziconotide

Based upon preclinical studies demonstrating the efficacy of ziconotide across a broad array of animal models of pain, clinical trials were initiated to determine the analgesic efficacy of ziconotide in pain-afflicted humans. The analgesic effectiveness of ziconotide in humans was characterized in 3 discrete randomized, double-blind, placebo-controlled studies [7,91,92]. Patients with severe chronic pain refractory to conventional pain management strategies were selected for participation. In each study, the route of drug delivery was an implanted intrathecal pump with continuous infusion. The large size and hydrophilicity of ziconotide prohibits movement across the blood-brain barrier to the spinally-located VGCC target following systemic administration [93]. Not only does intrathecal delivery increase the rate at which the drug reaches the site of action, but it also reduces the rate of ziconotide metabolism and excretion. Following intrathecal administration, ziconotide is distributed nearly exclusively in the cerebrospinal fluid and metabolized by proteolytic pathways only after transport into systemic circulation [94,95,96]. In two studies, a fast titration rate was selected for ziconotide infusion. The rapid infusion resulted in a 30%–53% reduction of pain, according to the Visual Analogue Scale of Pain Intensity, in ziconotide-treated patients with chronic non-malignant, or cancer- or AIDS-related pain. These patients also developed persistent cognitive and neuropsychiatric adverse effects which necessitated hospitalization [7,92]. At a slower dose titration rate, the severity of these adverse events was largely reduced, as was the analgesia; patients reported an average pain relief of only 14%. Yet some patients experienced complete pain relief with intrathecal ziconotide at slower infusion rates, suggesting this drug may be beneficial for a discrete population [91]. Notably, the analgesic efficacy of ziconotide was not limited by the development of tolerance, unlike analgesic opioids.

After extended Phase III clinical trials, intrathecal ziconotide was approved by the United States Food and Drug Administration for treatment of chronic refractory pain. With approval in 2004, ziconotide became the first venom-derived VGCC peptide inhibitor with clinical application [97]. However, because of the adverse events associated with fast dose titration of ziconotide, only slow titration regimens are approved for pain management. The reason for these adverse events and the relation to the rate of infusion is unclear [98]. Immune reactions to the injected peptide have been implicated as causative events for some ziconotide-related adverse effects, yet there is no evidence of hypersensitivity nor anaphylaxis in humans [28,99]. Rather, deleterious side effects might arise from complete inhibition of the N-type VGCCs [100]. In addition to expression in nociceptive nerve terminals, N-type VGCCs are widely distributed throughout the central nervous system where their function has been associated with hippocampal-dependent learning and memory [101,102,103]. Despite the marked selectivity of ziconotide for N-type channels over other VGCC isoforms, it is also possible that adverse events arise from off-target effects of ziconotide. *Ca_v_2.2*^−/^^−^ mice displayed reduced pain behavior in inflammatory pain models with very few observable adverse physiologic consequences, supporting the notion of ziconotide promiscuity [89].

### 4.4. Emerging Approaches to Reduce the Side Effect Profile of ω-Conotoxins

Although ziconotide is able to reduce pain and improve the quality of life in patients with neuropathic pain, the therapeutic window is narrow with severe, dose-limiting side effects [54]. There remains a critical need to identify N-type VGCC inhibitors with improved safety and alternative delivery methods for the management of neuropathic pain [39]. 

The 27-amino acid ω-conotoxin GVIA, isolated from *Conus geographus*, produces marked analgesia in nerve lesion and formalin-induced models of neuropathic pain [86]. Binding of this toxin to target N-type VGCCs blocked approximately 50% of Ca^2+^ influx through the channel, as opposed to the complete block achieved by ziconotide [100]. This feature made ω-conotoxin GVIA a desirable candidate for drug development; partial inhibition of Ca^2+^ influx could normalize pathologic hyperactivity of N-type VGCCs without perturbing VGCC-dependent homeostatic functions, effectively reducing side effects. It was later determined that the irreversible nature of GVIA inhibition may in fact result in more severe side effects [104]. Another ω-conotoxin, CVID from *Conus catus*, deemed the most potent of all N-type VGCC peptide blockers, showed promise as an efficacious analgesic in animal studies, but failed in clinical trials with, again, severe adverse effects [67,68,80,105].

The intrathecal route of delivery, though necessary for peptide drugs with centrally located sites of action, is in general unfavorable. Indeed, continuous infusion by intrathecal pump eliminates fluctuations in drug concentrations and reduces dose-related side effects associated with systemic administration. However, it is often one of the last choices for delivery of analgesics and comes with its own risks, including pump-associated infections. Implantation of an intrathecal pump is contraindicated in some medical disorders, such as bleeding diasthesis, and instances of concomitant treatment, such as intrathecal chemotherapy [54,106]. Contraindications limiting the application of intrathecal implants have driven the pursuit of a more convenient delivery method to increase the population of treatable patients. Even if peptides targeting N-type VGCCs elicited analgesia at the spinal level following oral or parenteral delivery methods, the presence of digestive enzymes or serum proteases, respectively, would shorten the drug’s half-life and stability in plasma [39]. Although peptide modification may slow proteolysis and increase bioavailability, there remains concern of altering the pharmacologic profile of the peptide or inadequately addressing the dosing limitations of ziconotide with these modifications [107]. To address this, pharmaceutical companies are actively pursuing small molecules derived from ω-conotoxins. One such molecule, NMED-160, advanced to Phase II clinical trials. Though the mechanism of NMED-160-induced analgesia mirrors that of ziconotide, the preeminent feature of this novel compound is its oral bioavailability. Despite appearing to be safe and well-tolerated in clinical trials, further development of NMED-160 was terminated without explanation [108]. A second small molecule, Z160, is currently progressing through Phase II clinical trials on the same premise of increasing oral bioavailability of ω-conotoxin derivatives.

## 5. Conclusions

The global impact of neuropathic pain and lack of adequate pharmacologic management places a great onus on the improvement of therapeutic strategies. Because neuropathic pain is often comorbid with other disease states, the pathogenesis of pain is complex and highly variable among individuals. Rather than treating the underlying mechanisms involved in the generation and maintenance of chronic pain, current first-line medications merely manage pain-related symptoms. The significant role of N-type VGCCs in synaptic transmission has directed therapeutic strategies towards correcting the electrophysiologic abnormalities of nociceptive neurons which underlie neuropathic pain symptoms. The ω-conotoxins, potent N-type VGCC inhibitors, solidified a role in pain management with the approval of ziconotide. However, the unfavorable side effect profile and inconvenience of intrathecal drug delivery continue to drive the improvement of our analgesic arsenal. The unique ability of marine derived toxins to target specific subtypes of ion channels coupled with their biological diversity may continue to contribute to this effort.
